# Chickpea and Chestnut Flours as Non-Gluten Alternatives in Cookies

**DOI:** 10.3390/foods10050911

**Published:** 2021-04-21

**Authors:** Marta Torra, Mayara Belorio, Manuel Ayuso, Marcio Carocho, Isabel C. F. R. Ferreira, Lillian Barros, Manuel Gómez

**Affiliations:** 1 Food Technology Area, College of Agricultural Engineering, University of Valladolid, 34071 Palencia, Spain; marta.torra295@gmail.com (M.T.); beloriom@gmail.com (M.B.); 2 Centro de Investigação de Montanha (CIMO), Instituto Politécnico de Bragança, Campus de Santa Apolónia, 5300-253 Bragança, Portugal; m.ayuso@ipb.pt (M.A.); mcarocho@ipb.pt (M.C.); iferreira@ipb.pt (I.C.F.R.F.)

**Keywords:** chickpea, chestnut, cookie, acceptability, nutritional quality

## Abstract

This study proposes the use of a mix composed of chickpea flour and chestnut flour in cookies, aiming to improve their acceptability. Cookie properties and nutritional value were also analysed. The gluten-free cookies were made by using different mixes of chickpea and chestnut flours (0:100, 25:75, 50:50, 75:25, 100:0). Dough rheology and cookie dimensions, texture, external colour and acceptability were evaluated. The presence of the chestnut flour increased the values of G’ and G”, but reduced the loss factor (tan δ) when compared with the doughs made with chickpea flour. Chestnut flour also decreased the diameter and the spread ratio of the cookies, while increasing the hardness and darkening of the cookies. Furthermore, adding chestnut to the flour mixture increased the nutritional quality of the cookies by adding unsaturated fatty acids and fibre. The use of reduced percentages of chestnut flour (25%) resulted in masking the off-flavour of the chickpea flour, which improved the cookie’s acceptability without significantly changing the dough rheology, cookie dimensions, hardness, or lightness.

## 1. Introduction

In recent years, there has been an increasing demand on the market for tasty and affordable foods containing nutritional and health benefits. Interest in the use of legume-based ingredients in food formulations is growing, mainly because of their nutritional properties [1], but also due to their positive functional properties [2], and the fact that they are gluten-free.

Legumes are known for their high protein content in comparison with vegetable products, and they are also rich in fibre and bioactive compounds, including enzyme inhibitors, lecithins, folates and phenolic compounds [3]. Legume consumption has positive effects on human health, namely protection from cardiovascular diseases, diabetes, cancer and obesity [1]. Interest in using legume flours in baked goods (both gluten-free and wheat-based) [2] has increased, despite difficulties as regards the use and consumption of legumes. These difficulties are related to the presence of antinutrients such as trypsin inhibitors, phytic acid and some non-digestible oligosaccharides which are related to digestive discomfort [4]. Furthermore, another problem of using legumes in baked goods is related to the presence of off-flavours which could reduce the acceptability of the final products by consumers [5]. 

Unfortunately, there is a lack of information in the literature as regards the reduction or elimination of these off-flavours in pulse-based food products, and traditional methods generally consist of adding sugars, salt, acids or aromas [6] to mask them.

Cookies could represent a good opportunity to use these flours due to their high level of acceptance by consumers and them having a comparatively long shelf-life. In general, this type of product does not contain a large amount of protein, and so a naturally rich protein flour could be almost exclusively used to improve the nutritional value of this product. Furthermore, a high gluten content and gluten strength may not be necessary, and sometimes not even desirable, to determine the good quality of cookies as regards their textural properties [7]. 

Research on the incorporation of different pulse flours, namely pea flours, in cookies has been conducted for a long time [8]. Still, these studies have focused on the use of bean flour [9,10], with only few reports on the use of chickpea flour [11,12]. In these studies, the percentage of chickpea flour in the formulation was very low (20%) or the results of consumer acceptability were not positive for high percentages of legume flour (greater than 40%). Most of the literature suggests that chickpeas are the most convenient legume to be used into baked goods [6]. Their addition has been shown to be useful as a substitute for other flours due to reductions in the acrylamide content in cookies and snacks [13].

Chestnut flour also has nutritional advantages, especially because of its high content of resistant starch [14] and bioactive compounds [15]. It also has a high sugar content, which could help to mask the off-flavors of chickpea flour, since consumers associate chestnut with sweet products, such as “marron glacees”. Chestnut flours have also been studied in the context of making cookies by using mixes with wheat flour [16], rice flour [17] or with a commercial gluten-free flour [18]. In terms of the latter, the ability of chestnut flour to improve oxidative stability during storage—due to its antioxidant properties—has been reported.

This study aims to demonstrate that sugar-snap cookies are a good product to apply chickpea flour in, since this allows for the production of gluten-free cookies with higher protein contents. Meanwhile, the use of chestnut flour can help to mitigate residual off-flavors. For this purpose, cookies were created with different mixes of chickpea flour (CPF) and chestnut flour (CNF) (100:0; 75:25, 50:50, 25:75, 0:100). Chemical analysis was performed to determine the nutritional composition, free sugars, and fatty and organic acids present in the flours and different cookie formulations. Additionally, dough rheology and cookie dimensions, texture and external colour were evaluated to determine the physical characteristics of the cookies. A consumer sensorial test was also carried out to evaluate the acceptability of the different cookies (colour, taste, odour, texture, and overall acceptability). 

## 2. Materials and Methods

### 2.1. Chemicals, Reagents, and Ingredients

The fibre enzymatic kit was obtained from Sigma-Aldrich (St. Louis, MO, USA), whereas all the chemicals and reagents were acquired from scientific common suppliers and were of (at least) analysis purity. Chickpea flour was supplied by Molendum Ingredients (Zamora, Spain) and chestnut flour was supplied by Sortegel (Sortes, Portugal). The other ingredients were acquired from retail stores and consisted of white sugar (AB Azucarera Iberica, Valladolid, Spain), 100% vegetable margarine (Argenta crema, Puratos, Barcelona, Spain), sodium bicarbonate (Manuel Riesgo S.A., Madrid, Spain) and potable water.

### 2.2. Cookie Preparation

Five different cookie samples were made using the following formulation (as g/100 g dough basis): flour (42.8), sugar (30.8), margarine (19.2), water (6.2) and sodium bicarbonate (0.9).

The ingredients of the five formulations were added in the same portions, except for the water, which was added to adjust flour moisture content to 15.0%, and the proportions of the flour. One cookie formulation was created with 100% of chickpea flour (100CPF), another with 100% of chestnut flour (100CNF) and the others were produced with flour mixes using different proportions of CPF and CNF: 75%CPF + 25%CNF (75CPF/25CNF), 50%CPF + 50%CNF (50CPF/50CNF) and 25%CPF + 75%CNF (25CPF/75CNF).

The margarine was heated in a microwave for 1 min at 1000 W, then, the melted margarine and the sugar were mixed in a Kitchen Aid 5KPM50 mixer (Kitchen Aid, Benton Harbor, MI, USA) using speed 4 for 3 min, scraping down the batter every 60 s. Then, the water was added and mixed at speed 4 for 2 min. At the end of this phase, flour and sodium bicarbonate were added and mixed at speed 2 for 2 min, stopping every 30 s to scrape down the batter. Afterwards, the dough was allowed to rest at 24 °C for 30 min, prior to laminating (6.00 mm gap) in pieces by a Salva L-500-J sheeter (Salva, Lezo, Spain) and cut with a circular cookie shaper with a diameter of 40 mm. Next, the cookies were baked at 185 °C for 14 min. Finally, the cookies were left at room temperature for 60 min and stored at 24 °C in plastic bags for further analysis. All the cookies were prepared in triplicate.

### 2.3. Chemical Analysis of Pulse Flours and Cookies

#### 2.3.1. Nutritional Profile

The nutritional values of the studied flours and cookies were determined based on their fat, protein, ash, carbohydrate, and dietary fibre contents, obtained following the AOAC official methods [19]. Crude fat was extracted from 3 g of sample in petroleum ether using a Soxhlet apparatus (AOAC 920.85) and expressed as g 100 g^−1^ of dry weight (DW). Crude protein was estimated as nitrogen content via the macro-Kjeldahl method (AOAC 978.04; *N* × 6.25 chestnut; *N* × 5.4 chickpea; *N* × 5.7 wheat). Crude protein was expressed as g 100 g^−1^ of DW. Ash content was determined by incineration at 550 ± 10 °C (AOAC 923.03) in a muffle (Lenton ECF 12/22, Hope Valley, UK). The total mineral content was expressed as g 100 g^−1^ of DW. The carbohydrate content was determined by the difference. The total available carbohydrates were expressed as g 100 g^−1^ of DW. Dietary fibre content was determined using the Megazyme kit based on the AOAC Method 985.29 and was expressed as g 100 g^−1^ of DW. Finally, the total energy was calculated using the European Parliament and Council Regulation No. 1169/2011 Formula (1):

*Energy* (*kcal 100 g*^−1^*DW*) = *4* × (*Protein* + *Carbohydrate*) *2* × (*Dietary fibre*) *9* × (*Fat*) (1)

#### 2.3.2. Soluble Sugars

Soluble sugars were determined using a High-Performance Liquid Chromatography (HPLC) system coupled to a refraction index (RI detector Knauer Smartline 2300, Berlin, Germany) detector. Chromatographic separation was achieved with a Eurospher100-5 NH2 column (5 μm, 4.6 × 250 mm, Knauer) as comprehensively described by Reis, Barros, Martins, Vasconcelos, Morales and Ferreira [20]. The samples (1 g) were extracted in a hydroethanolic (80:20 *v*/*v*) solution during 30 min at 80 °C. Melezitose was added as an internal standard (IS, Matreya, State College, PA, USA). The extracts were centrifuged (15,000× *g*, 10 min) and the supernatants were collected and concentrated under reduced pressure in a rotatory evaporator. Finally, the samples were defatted in ethyl ether (three times) and filtered using 0.2 µm Whatman’s nylon filters for HPLC analysis. The quantification was based on the RI signal response of each standard, depending on the IS method, and using calibration curves from commercial standards of each compound. The results were expressed in g 100 g^−^¹ of DW.

#### 2.3.3. Fatty Acids

Fatty acid analysis was performed via gas chromatography coupled to a flame ionisation detector (GC-FID). Fatty acids obtained were subjected to a transesterification procedure [21] and filtered with 0.2 µm Whatman’s nylon filters prior to their determination. Identification was performed by comparing the relative retention times of FAME (fatty acid methyl esters) peaks from samples with commercial standards (reference standard mixture 37; 47885-U, Sigma, St. Louis, MO, USA). The quantification was processed using the Clarity 4.0.1.7 software and expressed as a relative percentage of each fatty acid.

#### 2.3.4. Organic Acids

Organic acids were determined by ultra-fast liquid chromatography (UFLC, Shimadzu 20A series, Shimadzu Corporation, Kyoto, Japan) coupled with a photodiode array detector (PDA), which used 215 nm and 245 nm as preferred wavelengths. Samples were subjected to metaphosphoric acid extraction, as previously described by Barros, Pereira and Ferreira [22]. A SphereClone (Phenomenex, Hauppauge, NY, USA) reverse phase C18 column (5 μm, 250 × 4.6 mm i.d) was used at 35 °C to perform the chromatographic separation and the mobile phase used was sulphuric acid at 3.6 mM using a flow rate of 0.8 mL/min. Quantification of organic acids was done by comparing the area of their peaks with the commercial standards of each compound, using LabSolutions Multi LC-PDA software (Shimadzu Corporation, Kyoto, Japan). Quantification was achieved by comparing the area of organic acid peaks from the different samples with calibration curves obtained from commercial standards. The results were expressed in g 100 g^−1^ of DW.

### 2.4. Flour Properties

#### 2.4.1. Hydration Properties

Chickpea and chestnut flours were characterised by their hydration properties. Water holding capacity (WHC) is defined as the grams of water retained per gram of sample when not submitted to stress. WHC was evaluated following the standard method [23]. An amount of 5 g (±0.1 g) of flour sample was dispersed in 100 mL of distilled water in a test tube and kept at room temperature to hydrate for 24 h. The excess water was removed, and the hydrated solid was weighed. Swelling volume (SV) was obtained through the relation between the total volume of the swollen sample and the original dry weight of the sample. Both SV and WHC were evaluated three times.

#### 2.4.2. Particle Size

Particle size distribution was determined using a Mastersizer 3000 particle size analyser (Malvern Instruments, Malvern, United Kingdom). Values of D[4,3], which represents the equivalent spherical diameter of the particles, and D(90), which represents the maximum particle diameter, below which 90% of the sample falls, respectively, were obtained. Measurements were carried out in triplicate.

### 2.5. Dough Characteristics

Rheological measures of the cookie dough were conducted using a controlled strain rheometer (Haake RheoStress 1, Thermo Fisher Scientific, Schwerte, Germany) at 25 °C. Circular dough pieces (3 mm height and 60 mm diameter) were placed on titanium parallel-serrated plate geometry PP60 Ti and compressed with a gap of 3 mm. Vaseline oil (Panreac, Panreac Química SA, Castellar del Valles, Spain) was added to the exposed sample surface to avoid drying during the analysis. For the identification of the LVR (linear viscoelastic region), the first measurement involved a strain sweep test performed a constant frequency (1 Hz) in the range of 0.1–100 Pa. The stress value obtained from the first test was used in a frequency sweep test with a frequency range from 10 to 0.1 Hz, to obtain the values of the elastic modulus (G’ (Pa)), viscous modulus (G” (Pa)), complex modulus (G* (Pa)) and the loss factor (tan δ) (G”/G’). Every sample was evaluated twice.

### 2.6. Cookie Characteristics

Cookie characteristics were evaluated 1 day after baking. Six cookies of each batch were evaluated. Cookie dimensions were obtained using a calliper. The diameter of each cookie was measured three times, perpendicularly, to obtain the average diameter. To obtain the spread factor, the average diameter was divided by the thickness. The surface colour was measured using a Colorimeter PCE-CSM 1 (Southampton, UK) with the D65 standard illuminant, LED light source type, blue light excitation and a 10º standard observer. The results were expressed in the CIE L*a*b* colour space. Measurements were made at the centre of the upper surface. The texture of the cookies was measured by a “three-point bending test” with the use of a TA-XT2 texture analyser (Stable Microsystems, Surrey, UK) running the “Texture Expert” software. The conditions used during the experiment were as follows: supports 30 mm apart, a 20 mm probe travel distance, a trigger force of 5 g and a test speed of 2.0 mm/s. The hardness (N), which is the maximum force used to break the cookies, was obtained.

### 2.7. Acceptance Test

A sensory analysis was performed in two parts. In the first part, volunteers evaluated the cookies acceptability according to 5 attributes (visual appearance, odour, texture, taste, and overall acceptability) using a hedonic scale from 1 (extremely dislike) to 9 (extremely like). The second part aimed to quantify how many consumers would identify the flavour of chickpea or chestnut in the cookies. They received a paper with a table containing six different ingredients (wheat, chickpea, banana, corn, beans, and chestnut) and they were instructed to evaluate the perception of these flavours in each sample by using a scale composed by 0 (“I do not taste this flavour”), 1 (“I taste this flavour and it is slight”) and 2 (“I taste this flavour and it is intense”). 

These analyses were carried out according to the protocol previously approved by the Committee of Tests and Research from the Hospital Rio Carrión (Palencia, Spain). The tests were made in a tasting room at the university with appropriate light and available potable water. The volunteers were a total of 93 usual cookies consumers whose range of age 18 to 64 years of age. Samples were analysed one week after baking. The five cookies, one of each type, were placed on white plastic plates coded with four-digit numbers and served in random order.

### 2.8. Statistical Analysis

Statistical tests for the chemical analysis were using SPSS Statistics (IBM SPSS Statistics v. 25., IBM Corp, Armonk, NY, USA). The results were analysed using a one-way analysis of variance (ANOVA one-way) followed by Tukey’s HSD post hoc test for the cookie samples, and a Student’s *t*-test for the flour samples after confirming their homoscedasticity. The statistical analysis for the acceptance tests, flour properties, dough, and cookie characteristics were performed using Statgraphics Plus 5.1 software (Statpoint Inc., Warrenton, VA, USA). Differences between the parameters of the different formulations were studied by analysis of variance (One-way ANOVA) using Fisher’s least significant difference test as a post hoc to determine the significant differences between the respective means. All analyses were performed using 95% confidence intervals and represented as average ± SD (standard deviation).

## 3. Results and Discussion

### 3.1. Flour Analysis

#### 3.1.1. Nutritional Profile and Chemical Analysis

The nutritional profiles of the flours, the soluble sugars, organic acids, and fatty acids are shown in Table 1. The CPF showed statistically significant higher values of fat, protein, total dietary fibre, and energy, while chestnut showed higher values of ash and carbohydrates. The high concentration of carbohydrates in chestnut flour can be explained by the high starch content of this nut [24]. 

In terms of the soluble sugars, glucose and trehalose were only detected in chestnut flour, which also recorded higher values of fructose and sucrose, and consequently total sugars.

Four organic acids were identified, namely oxalic, quinic, malic and fumaric acids. Quinic was only found in CNF, while malic was only found in chickpea flour. Oxalic was detected in a higher quantity in CPF and fumaric was only found in traces. Overall, chickpea flour showed the highest organic acid concentration.

Chestnut flour presented higher quantities of fatty acids, mainly polyunsaturated fatty acids, than chickpea flour. However, chickpea flour exhibited a greater variety of fatty acids and a higher quantity of monounsaturated fatty acids than chestnut flour. The most abundant individual fatty acid in the CPF was oleic acid, while linoleic was the most abundant in the CNF, which did not present any monounsaturated fatty acids, but rather 48% of saturated fatty acids, while chickpea showed 45% and 43% of monounsaturated fatty acids, and 45% of saturated fatty acids, making it a healthier flour. Chickpea and chestnut are good sources of nutritionally important unsaturated fatty acids. Jukanti, Gaur, Gowda and Chibbar [25] also described the existence of linoleic, oleic, and palmitic, while oleic acid appears in a high concentration in some chickpea cultivars. Pulses of chickpea, bean, lentil, and others are an important source of vegetable proteins and fibre, and their consumption is essential for human health since they may comprise several benefits, including reduction in the risk of cardiovascular diseases, cancer, type-2 diabetes, hypertension, gastrointestinal disorders, and cholesterol [10,26,27]. Thus, pulses such as chickpea have been used in several composite flours and functional foods, contributing to improving the nutritional quality of the resulting products [28]. CNF contains a low amount of fat, desirable dietary fibre, and good mineral contents. It was added to the cookies to increase their organoleptic and nutritional quality.

#### 3.1.2. Flour Properties

**Figure 1:**
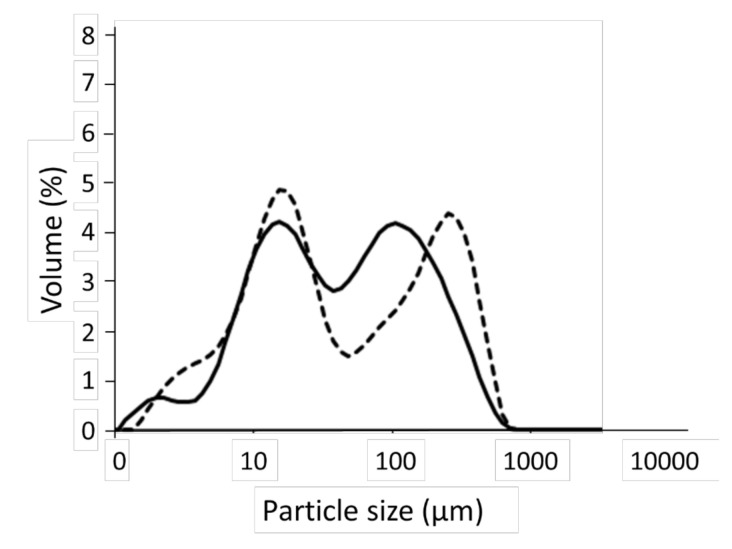
Particle size distribution of the chestnut (whole line) and chickpea (dashed line) flours.

Figure 1 shows the particles size distributions of CPF and CNF, which were similar, and both flours had a bimodal distribution with two markable peaks. However, while the peaks of the thin particles were similar, those of the coarse particles had a higher particle size compared to CPF. This result generated high values of D(90) and D[4,3] to CPF (Table 2).

As regards the hydration properties (WHC and SV), no significant differences were found between CPF and CNF, or between these samples and the different flour mixes (Table 2). Values of between 1.97 and 2.17 g/g were obtained for WHC, while SV ranged from 2.60 and 2.90 mL/g. CNF presented small particle sizes and different authors found that the smaller the particle size, the greater the hydration properties due to a great surface area [29,30]. However, flours with a high protein content, such as CPF, had an increase in hydration properties [7,31]. Therefore, both phenomena could be compensated, so no significant differences were observed. 

#### 3.1.3. Dough Characteristics

The rheology of the dough is a characteristic that requires important attention when preparing a cookie formulation. In fact, if the dough is too soft or too firm it is not easy to manipulate, thus, the dough must be sufficiently cohesive to remain united during the process and to be easily laminated, without being so sticky that is becomes attached to the rolling mill. According to Belorio et al. [30] to make a cookie dough that is cohesive and laminable, it is necessary that it contains a minimum proportion of small-sized particles, which occurs in both chickpea and chestnut flours. 

The results obtained from the rheological analysis are shown in Table 2. All doughs showed a higher value of storage modulus (G’) than loss modulus (G”), which suggests a solid elastic-like behaviour [32]. The viscoelastic parameter, tan δ, which indicates the contribution of elastic and viscous components of the dough, was less than 1 for all samples.

The G’, G” and G* values showed a tendency to increase due to an increment in the percentages of chestnut flour in the mixes. However, no significant differences were observed for G’ when doughs were created with mixes containing chickpea flour. As regards G” and G*, no significant differences were found for doughs containing up to 50% CPF. Values of tan δ reduced with increasing percentages of CNF in the formulations. Demirkesen et al. [17] found a similar tendency when CNF substituted rice flour in cookies dough formulations. Authors attributed this tendency to the high-fibre content of CNF, higher than 9% [15] and to the low fibre content of rice flour, which is also a characteristic of CPF [1]. In addition, these authors associated this difference with the high-water absorption capacity of fibres. However, in our study, there were no significant differences between the hydration properties of the different samples and the high protein content of CPF can also increase the G’ and G” values of the doughs [31]. Another explanation for this result could be the high sugar content of CNF, since the sugar competes with other ingredients for the water, which increases the cookie dough viscosity [33]. Thus, the observed effect is a combination between the distinct influences that flour components produce over the dough rheology, without forgetting the influence of particles sizes [30].

### 3.2. Cookie Analysis

#### 3.2.1. Cookies Nutritional Profile and Chemical Analysis

The cookies, baked with varying concentrations of CPF and CNF, also underwent a nutritional profile analysis as well as individual compounds such as soluble sugars, organic acids and fatty acids, and are presented in Table 3. As regards the nutritional profile, crude fat did not show any significant difference between all samples, while dietary fibre was higher in the cookies with 100% CPF. Thus, all tested cookies can be considered as containing “high dietary fibre” according to Regulation (EU) No. 432/2012 and Regulation (EC) No. 1924/2006, providing more than 6 g 100g^−1^ of DW. As expected, the cookies with CNF showed a higher quantity of carbohydrates, while the cookies with CPF had a higher concentration of proteins and fibre. Once again trehalose and glucose were only detected in the cookies with 100% CNF, and the chestnut cookies had the highest soluble sugar quantity with significant differences in comparison with all other samples. In terms of the oxalic acids, the same ones were identified in the cookies, and thus, once again, fumaric acid was only detected in trace amounts or was not detected at all, while malic acid was only detected in the cookies with higher concentrations of CPF and quinic acid in samples with CNF. Oxalic acid was found in higher amounts in CPF cookies, as expected. As regards the fatty acids, contrarily to the flours, the most abundant fatty acid in all cookies was palmitic acid due to its high presence in the margarine that was used to make the cookies. Still, the highest amounts of saturated fatty acids were sought for all the cookies with CPF, while the unsaturated fatty acids were quite similar in all cookies, exception made to the monounsaturated fatty acids of the cookies made with 25% CPF and 75% CNF.

#### 3.2.2. Cookie Physical Parameters

As can be seen in Table 4, the bigger the CNF percentage, the smaller the spread ratio. This parameter is considerably influenced by cookie height, which was bigger in cookies with 50% or more CPF, when compared with cookies with 25% or less CPF. The difference between cookie height was smaller and the smaller height of cookies made with CNF compared to cookies created with mixes of flours is highlighted. During the first part of baking, fat melts and sugars reduce the dough viscosity, allowing for the relaxation and expansion of the dough [7].

In general, most studies find correlations between the dough rheology and the spread factor, so that when values of G’ and G” are increased, the diameter of the cookies is reduced [34,35], similar to the results found herein. Demirkesen [17] found a reduction in cookie diameter when the amount of CNF was increased compared with the amount of rice flour in the formulation, which coincides with the results of this study. However, the incorporation of CPF did not have a clear effect on these parameters and while the spread factor reduced in flour mixes of amaranth or buckwheat, it did not decrease in mixes with wheat flour [12]. Therefore, according with the results of this study, the reduction effect of the spread factor seemed to be bigger for CNF than for CPF and this could be related to the dough rheology, which was discussed in the previous section. 

Additionally, there is a tendency for the hardness to increase as the content of chestnut flour increases. This finding is also consistent with the result of Dokić et al. [32], who showed an increase in hardness values of wheat cookie samples when a high amount of wheat flour was replaced with CNF. Similar results were found in gluten-free cookies when rice flour was substituted by CNF at percentages above 40% [17]. Nevertheless, a small addition of CNF generated a reduction in the hardness of rice flour cookies. These differences could be related to the influence of flour particle size distribution, which was not evaluated or to the cookie formulation and its greater amount of fat than sugar, which is contrary to the formulation used in this study. In general, cookies with small spread factor and with a compact structure present high hardness [11,31,34], which was also confirmed herein. Demirkesen [17] assigned this increase in hardness to the high sugar content of CNF, which is related to crystallization phenomenon after cooling and with sugar caramelization. Jiang, Liu, Bhandari and Zhou [36] observed that harsh caramelization conditions, such as those produced during baking, can increase the glass transition temperature of sugars, which can influence cookie texture. 

**Figure 2:**
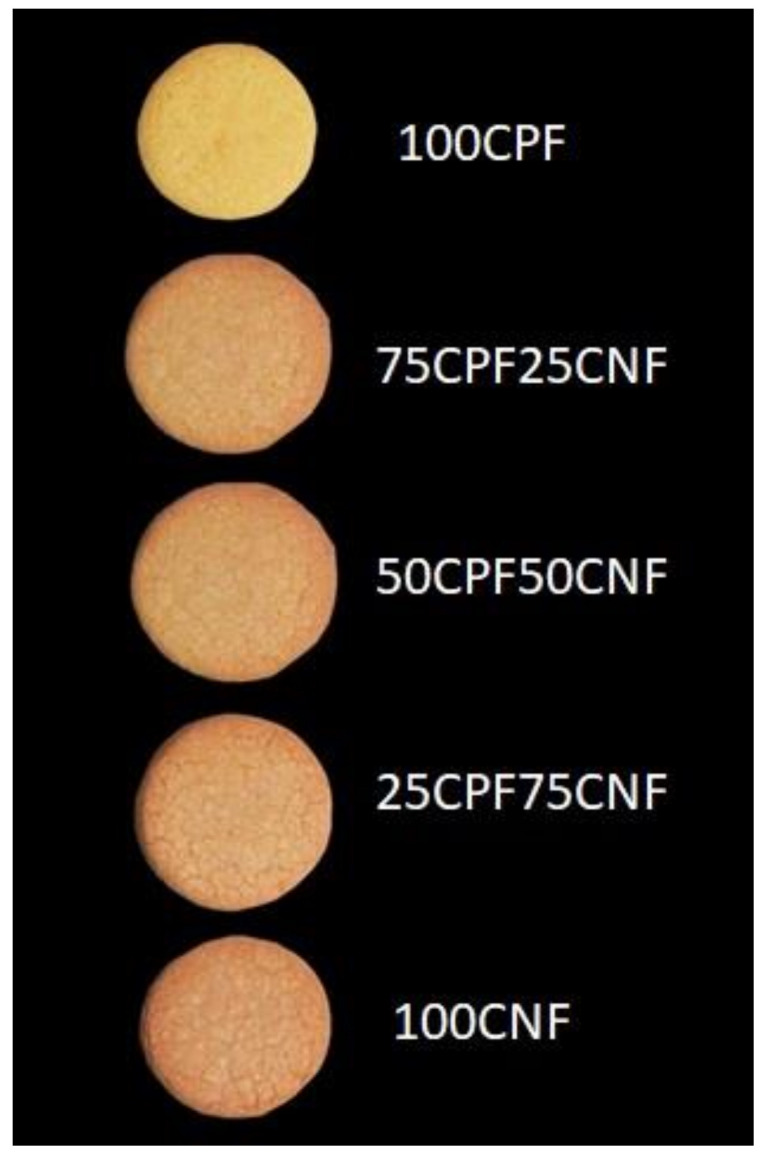
Cookies produced with chestnut and chickpea flour.

Figure 2 shows the surface colour of the cookies, which is one of the important quality factors that affects the acceptability of the final product by the consumer. The lightness (L*) (Table 4) decreased with an increase in the amount of CNF. Parameters a* also increased (more reddish) and b* decreased (less yellowish) as the amount of CNF increased. Other authors found similar results when substituting wheat flour [36] or rice flour [17] for CNF. Nevertheless, in addition to a decrease in L* and an increase in a*, these authors also found an increase in b*. These differences could be related to the different formulations used in these studies. The colour of cookies mainly depends on Maillard reactions, which occur between proteins and reducing sugars, and on sugar caramelization [37]. In addition, the original colour of flours can also influence the colour of the cookie. CPF and CNF have light tones of colour, so the darkness of cookies is related to the reactions produced during baking. Although CPF has a high protein content which could influence the Maillard reactions, the high sugar content of CNF could influence the caramelization reactions. This reaction depends on the sugars type but, in general, it occurs at temperatures above of 120 °C [38], which is inferior to those produced during cookie baking. It is important to consider that, even if the sugar content depends on the kind of sugar, it is often above of 14% in CNF, and sucrose is the major sugar [39].

#### 3.2.3. Sensorial Analysis

The results of cookie acceptability are shown in Table 4. All cookies obtained values of global acceptability higher than five, on a scale range of one to nine, and the best evaluated cookie presented 6.77. This value is superior to the evaluation of wheat cookies [33] or gluten-free cookies [34], which were evaluated in other studies using the same formulation. Cookies made with flour mixes presented a better overall evaluation with respect to cookies made with only CNF. The increasing percentage of CPF in flour mixes indicated a trend to improve the acceptability of cookies. In fact, the best evaluated cookie was the one with high percentage of CPF (75%), although it did not present significant differences with respect to the other cookies elaborated with flour mixes. Increasing amounts of CNF generated worse values of cookie appearance, possibly due to their dark colour and small dimensions. Thus, the mouthfeel (taste and texture) was the parameter that most influenced the overall acceptability of cookies. Other authors observed negative effect on acceptability when incorporating CPF in wheat flour cookies [12]. It is known that legume flours have a negative effect on the acceptability of cookies due to the off-flavors, which can be reduced by increasing the amount of sugar or adding flavours in the formulation [6]. The incorporation of CNF helps to produce this effect due to its high sugar content and to its flavour. However, large amounts of CNF are negative because of the high level of hardness and the darkness of cookies, which influence not only the cookie aspect, but are often related to the generation of compounds during baking that reduce cookie acceptability [40]. This negative effect of CNF was previous observed by Šoronja-Simović et al. [16] in wheat cookies. 

Consumers were asked about the identification of different flavours in the cookies, among different options. They then had to establish if this was slight or intense (data not shown). In the case of CPF cookies, more than 15% of the consumers identified the chickpea flavour and they qualified it as intense in 90% of the cases. The identification of chestnut flavour was smaller (10%) and it was identified as intense in 66% of the cases. Nevertheless, the number of consumers that identified the flavours in cookies produced with flour mixes was even smaller and in most of cases it was qualified as a slight flavour. The percentage of consumers that correctly identified the flavour mix was not greater than 3% for all the cases. In samples with 75% of CPF, 12% of the consumers identified the chickpea flavour, but only 40% qualified it as intense. In mixes made with 50% of CPF and CNF, only 5% of the consumers identified an intense flavour of chickpea. Thus, the use of CNF contributed to masking and mitigating the chickpea flavour, which can contribute to improve cookie quality.

## 4. Conclusions

The combination of CPF and CNF can generate cookies with better overall acceptability than cookies produced with only one of these flours. The incorporation of CNF generated cookies with a small diameter, that were darker and harder. However, in small percentages, it contributed to improving the acceptability of cookies produced with CPF. Beyond this, the use of CPF and CNF allow for the production of cookies considered as containing “high in dietary fibre”. Finally, the use of reduced percentages of chestnut flour (25%) improved the acceptability of chickpea cookies without significantly changing dough rheology, cookies dimension, hardness, or the lightness of these cookies.

## Figures and Tables

**Table 1:** Centesimal composition, soluble sugars, organic acids (g/100 g dw) and fatty acids (%) of the chickpea and chestnut flour samples.

Nutritional Value	CPF ¹	CNF ²
Crude Fat	4.6 ± 0.3 *	2.40 ± 0.03
Protein	19.9 ± 0.2 *	8.0 ± 0.3
Ash	6.0 ± 0.5	7.7 ± 0.3 *
Carbohydrates	53.8 ± 0.3	71 ± 1 *
Total dietary fibre	15.7 ± 0.1 *	10.8 ± 0.5
**Soluble Sugars**		
Fructose	1.0 ± 0.1	1.4 ± 0.1 *
Glucose	nd	1.5 ± 0.2
Sucrose	4.1 ± 0.1	16.2 ± 0.6 *
Trehalose	nd	0.7 ± 0.1
**Total Sugars**	5.1 ± 0.2	20 ± 1 *
**Organic acids**		
Oxalic acid	0.046 ± 0.002 *	0.016 ± 0.002
Quinic acid	nd ^3^	0.015 ± 0.003
Malic acid	0.13 ± 0.02	nd ^3^
Fumaric acid	tr ^4^	nd ^3^
**Total Organic Acids**	0.18 ± 0.02 *	0.031 ± 0.001
**Fatty Acids**		
C10:0 ^5^	3.94 ± 0.02	nd ^3^
C14:0 ^6^	4.24 ± 0.01	nd ^3^
C15:0 ^7^	3.82 ± 0.01	nd ^3^
C16:0 ^8^	16.22 ± 0.01	26.54 ± 0.03 *
C16:1 ^9^	3.93 ± 0.03	nd ^3^
C17:0 ^10^	3.81 ± 0.02	nd ^3^
C18:0 ^11^	4.80 ± 0.01	22.23 ± 0.02 *
C18:1n9c ^12^	35.25 ± 0.14	nd ^3^
C18:2n6c ^13^	7.20 ± 0.01	28.37 ± 0.01 *
C18:3n3 ^14^	4.45 ± 0.02	22.86 ± 0.02 *
C20:0 ^15^	4.22 ± 0.01	nd ^3^
C21:1 ^16^	4.17 ± 0.01	nd ^3^
C22:0 ^17^	3.940 ± 0.003	nd ^3^
Total SFA ^18^	45.01 ± 0.09	48.78 ± 0.01 *
Total MUFA ^19^	43.4 ± 0.1	nd ^3^
Total PUFA ^20^	11.65 ± 0.01	51.22 ± 0.01

^1^ Chickpea flour; ^2^ Chestnut flour; ^3^ Not detected; ^4^ Trace amount; ^5^ Capric acid; ^6^ Myristic acid; ^7^ Pentadecylic acid; ^8^ Palmitic acid, ^9^ Palmitoleic acid; ^10^ Margaric acid, ^11^ Stearic acid, ^12^ Oleic acid; ^13^ Linoleic acid; ^14^ α-Linolenic acid; ^15^ Arachidic acid; ^16^ Heneicosylic acid, ^17^ Behenic acid. ^18^ SFA: saturated fatty acids; ^19^ MUFA: monounsaturated fatty acids. ^20^ PUFA: polyunsaturated fatty acids. In each row, * represents significant statistical differences between flours *p* < 0.05.

**Table 2:** Flour properties and rheological characteristics of cookie doughs of the chickpea and chestnut mixtures.

	100CPF ^1^	75CPF25CNF	50CPF50CNF	25CPF75CNF	100CNF ^2^
WHC ^3^ (g/g)	2.08 ± 0.14 ^a^	2.13 ± 0.06 ^a^	2.17 ± 0.05 ^a^	2.03 ± 0.14 ^a^	1.97 ± 0.07 ^a^
SV ^4^ (mL/g)	2.86 ± 0.31 ^a^	2.80 ± 0.00 ^a^	2.90 ± 0.14 ^a^	2.60 ± 0.20 ^a^	2.60 ± 0.20 ^a^
D(4;3) ^5^	113 ± 4 ^b^	nd ^11^	nd ^11^	nd ^11^	80 ± 8 ^a^
D(90) ^6^	329 ± 13 ^b^	nd ^11^	nd ^11^	nd ^11^	211 ± 23 ^a^
G’ (Pa) ^7^	100,255 ± 13 ^a^	100,540 ± 4 ^a^	115,060 ± 17 ^a^	249,100 ± 1 ^a^	685,200 ± 5 ^b^
G”(Pa) ^8^	42,660 ± 1344 ^a^	38,075 ± 3642 ^a^	38,055 ± 6131 ^a^	65,185 ± 1435 ^b^	101,695 ± 4391 ^c^
Tan δ ^9^	0.45 ± 0.02 ^c^	0.40 ± 0.01 ^bc^	0.33 ± 0.01 ^abc^	0.28 ± 0.01 ^ab^	0.26 ± 0.11 ^a^
G* ^10^	109,250 ± 10,536 ^a^	108,250 ± 5162 ^a^	121,250 ± 23,405 ^a^	257,900 ± 1131 ^b^	656,300 ± 88,247 ^c^

^1^ Chickpea flour; ² Chestnut flour; ³ Water holding capacity; ^4^ Swelling volume; ^5^ Equivalent spherical diameter of the particles; ^6^ Maximum particle diameter below which 90% of the sample fall; ^7^ Storage modulus; ^8^ Loss modulus; ^9^ Loss factor; ^10^ Complex modulus; ^11^ Not detected. Values with different letters in the same row represent statistically significant differences (*p* < 0.05).

**Table 3:** Centesimal composition, soluble sugars, organic acids, (g/100 g DW) and fatty acids (%) of the cookies made with different amount of chickpea and chestnut flours.

**Nutritional Value**	**100CPF ^1^**	**75CPF25CNF**	**50CPF50CNF**	**25CPF75CNF**	**100CNF ^2^**
Crude Fat	15.71 ± 0.03 ^a^	15.6 ± 0.5 ^a^	15.6 ± 0.2 ^a^	15.2 ± 0.2 ^a^	15.2 ± 0.3 ^a^
Proteins	9.8 ± 0.5 ^a^	8.0 ± 0.2 ^b^	6.9 ± 0.4 ^c^	5.97 ± 0.01 ^d^	3.9 ± 0.4 ^e^
Ash	5.0 ± 0.5 ^a^	1.8 ± 0.1 ^b^	4.9 ± 0.5 ^a^	2.5 ± 0.1 ^b^	5.5 ± 0.4 ^a^
Carbohydrates	58 ± 1 ^c^	64 ± 1 ^b^	63 ± 1 ^b^	68 ± 1 ^a^	66 ± 0.9 ^a^
Total dietary fibre	11.2 ± 0.1 ^a^	10.2 ± 0.1 ^b^	9.6 ± 0.6 ^bc^	8.7 ± 0.3 ^c^	9.0 ± 0.1 ^c^
Energy (kcal/100 g dw)	435 ± 2 ^b^	449 ± 2 ^a^	439 ± 3 ^b^	450 ± 1 ^a^	434 ± 1 ^b^
**Soluble Sugars**	**100CPF**	**75CPF25CNF**	**50CPF50CNF**	**25CPF75CNF**	**100CNF**
Fructose	3.0 ± 0.1 ^a^	2.0 ± 0.1 ^bc^	1.8 ± 0.5 ^c^	1.4 ± 0.1 ^c^	2.9 ± 0.5 ^ab^
Glucose	nd ^3^	nd ^3^	nd ^3^	nd ^3^	2.0 ± 0.6
Sucrose	21.5 ± 0.8 ^b^	14.4 ± 0.4 ^c^	16.3 ± 0.9 ^c^	19.7 ± 0.5 ^b^	35 ± 2 ^a^
Trehalose	nd ^3^	nd ^3^	nd ^3^	nd ^3^	1.3 ± 0.2
**Total Sugars**	24.5 ± 0.9 ^b^	16.3 ± 0.3 ^d^	18.0 ± 0.4 ^cd^	21 ± 1 ^c^	41 ± 3 ^a^
**Organic acids**					
Oxalic acid	0.094 ± 0.001 ^b^	0.115 ± 0.005 ^a^	0.037 ± 0.002 ^c^	0.035 ± 0.002 ^c^	0.004 ± 0.001 ^d^
Quinic acid	nd ^3^	nd ^3^	nd ^3^	0.118 ± 0.005 ^a^	0.047 ± 0.003 ^b^
Malic acid	0.155 ± 0.003 ^a^	0.123 ± 0.002 ^b^	0.10 ± 0.01 ^c^	nd ^3^	nd ^3^
Fumaric acid	tr ^4^	tr ^4^	tr ^4^	tr ^4^	nd ^3^
**Total Organic Acids**	0.25 ± 0.07 ^a^	0.238 ± 0.004 ^a^	0.14 ± 0.01 ^b^	0.15 ± 0.01 ^b^	0.051 ± 0.002 ^c^
**Fatty Acids**	**100CPF**	**75CPF25CNF**	**50CPF50CNF**	**25CPF75CNF**	**100CNF**
C12:0 ^5^	0.33 ± 0.01 ^a^	0.308 ± 0.001 ^ab^	0.34 ± 0.01 ^a^	0.29 ± 0.03 ^b^	0.30 ± 0.01 ^ab^
C14:0 ^6^	1.04 ± 0.03 ^a^	1.02 ± 0.01 ^a^	1.06 ± 0.01 ^a^	0.9 ± 0.1 ^b^	0.98 ± 0.03 ^ab^
C15:0 ^7^	nd ^3^	nd ^3^	nd ^3^	nd ^3^	0.06 ± 0.01
C16:0 ^8^	43.8 ± 0.4 ^bc^	44.2 ± 0.1 ^b^	44.40 ± 0.02 ^b^	43.5 ± 0.4 ^c^	45.4 ± 0.1 ^a^
C16:1 ^9^	0.17 ± 0.01 ^a^	0.153 ± 0.001 ^b^	0.158 ± 0.001 ^b^	0.155 ± 0.002 ^b^	0.129 ± 0.002 ^c^
C17:0 ^10^	0.12 ± 0.01 ^a^	nd ^3^	nd ^3^	nd ^3^	0.12 ± 0.01 ^a^
C18:0 ^11^	4.74 ± 0.04 ^a^	4.56 ± 0.02 ^b^	4.56 ± 0.01 ^b^	4.50 ± 0.03 ^b^	2.85 ± 0.01 ^c^
C18:1n9c ^12^	32.8 ± 0.2 ^b^	32.93 ± 0.06 ^b^	33.09 ± 0.01 ^b^	34.1 ± 0.3 ^a^	32.7 ± 0.1 ^b^
C18:2n6c ^13^	15.5 ± 0.1 ^bc^	15.83 ± 0.04 ^a^	15.397 ± 0.003 ^c^	15.6 ± 0.2 ^abc^	15.67 ± 0.03 ^ab^
C18:3n3 ^14^	0.452 ± 0.004 ^b^	0.450 ± 0.002 ^b^	0.442 ± 0.001 ^b^	0.43 ± 0.01 ^b^	0.56 ± 0.04 ^a^
C20:0 ^15^	0.33 ± 0.01 ^b^	0.289 ± 0.002 ^c^	0.286 ± 0.001 ^c^	0.282 ± 0.001 ^c^	0.424 ± 0.001 ^a^
C20:1 ^16^	0.146 ± 0.002 ^a^	0.116 ± 0.001 ^b^	0.116 ± 0.001 ^b^	0.116 ± 0.003 ^b^	0.10 ± 0.02 ^b^
C20:2 ^17^	0.40 ± 0.01 ^b^	nd ^3^	nd ^3^	nd ^3^	0.436 ± 0.002 ^a^
C22:0 ^18^	nd ^3^	nd ^3^	nd ^3^	nd ^3^	0.193 ± 0.001
C22:1 ^19^	0.19 ± 0.01 ^a^	0.145 ± 0.003 ^c^	0.155 ± 0.001 ^b^	0.149 ± 0.001 ^bc^	nd ^3^
C24:0 ^20^	nd ^3^	nd ^3^	nd ^3^	nd ^3^	0.124 ± 0.001
Total SFA ^21^	50.4 ± 0.3 ^a^	50.4 ± 0.1 ^a^	50.642 ± 0.001 ^a^	49.5 ± 0.5 ^b^	50.05 ± 0.04 ^b^
Total MUFA ^22^	33.3 ± 0.2 ^bc^	33.3 ± 0.1 ^bc^	33.52 ± 0.01 ^b^	34.5 ± 0.3 ^a^	32.9 ± 0.1 ^c^
Total PUFA ^23^	16.4 ± 0.1 ^bc^	16.28 ± 0.04 ^a^	15.838 ± 0.004 ^c^	16.0 ± 0.2 ^abc^	16.7 ± 0.1 ^ab^

^1^ Chickpea flour; ^2^ Chestnut flour; ^3^ Not detected; ^4^ Trace amount; ^5^ Lauric acid; ^6^ Myristic acid, ^7^ Pentadecylic acid; ^8^ Palmitic acid; ^9^ Palmitoleic acid, ^10^ Margaric acid; ^11^ Stearic acid; ^12^ Oleic acid; ^13^ Linoleic acid; ^14^ α-Linolenic acid; ^15^ Arachidic acid; ^16^ Eicosenoic acid; ^17^ Eicosadienoic acid; ^18^ Behenic acid; ^19^ Erucic acid; ^20^ Lignoceric acid. ^21^ Saturated fatty acids; ^22^ Monounsaturated fatty acids; ^23^ Polyunsaturated fatty acids. Different letters in the same row refer to statistically significant differences at *p* < 0.05 according to Tukey’s HSD test.

**Table 4:** Physical characteristics and sensory acceptability of the cookies made with different mixes of chickpea and chestnut flours.

	100CPF ^1^	75CPF25CNF	50CPF50CNF	25CPF75CNF	100CNF ^2^
**Physical characteristics**					
Diameter (mm)	49.79 ± 2.31 ^b^	50.69 ± 1.25 ^b^	49.25 ± 0.28 ^b^	45.89 ± 0.36 ^a^	43.37 ± 0.61 ^a^
Thickness (mm)	9.26 ± 0.32 ^ab^	9.57 ± 0.16 ^b^	9.45 ± 0.21 ^b^	9.65 ± 0.10 ^b^	9.01 ± 0.04 ^a^
Spread ratio	5.38 ± 0.08 ^c^	5.31 ± 0.09 ^bc^	5.21 ± 0.10 ^b^	4.76 ± 0.02 ^a^	4.82 ± 0.07 ^a^
Hardness (N)	56.0 ± 4.1 ^ab^	51.9 ± 1.9 ^a^	56.1 ± 0.3 ^ab^	61.2 ± 6.4 ^bc^	64.5 ± 2.8 ^c^
Gradient (N/s)	183.5 ± 26.9 ^a^	202.7 ±11.9 ^ab^	189.7 ± 25.3 ^a^	229.74 ± 1.1 ^b^	236.1 ± 12.5 ^b^
**Colour parameters**					
L*	52.70 ± 0.80 ^cd^	54.89 ± 4.39 ^d^	45.87 ± 2.12 ^bc^	37.67 ± 0.41 ^a^	43.15 ± 4.89 ^ab^
a*	8.45 ± 0.67 ^a^	10.70 ± 0.82 ^b^	9.79 ± 0.48 ^b^	10.53 ± 0.06 ^b^	12.54 ± 0.33 ^c^
b*	31.11 ± 0.85 ^c^	29.42 ± 1.39 ^c^	24.69 ± 1.43 ^b^	21.41 ± 0.08 ^a^	22.50 ± 1.73 ^ab^
**Sensory acceptability**					
Appearance	7.32 ± 1.54 ^d^	7.13 ± 1.49 ^cd^	6.66 ± 1.46 ^b^	6.73 ± 1.64 ^bc^	5.94 ± 1.96 ^a^
Odour	6.01 ± 1.68 ^ab^	6.19 ± 1.57 ^ab^	6.44 ± 1.49 ^b^	6.29 ± 1.54 ^b^	5.83 ± 1.68 ^a^
Texture	5.85 ± 1.78 ^b^	6.36 ± 1.56 ^c^	5.98 ± 1.58 ^bc^	5.84 ± 1.68 ^b^	4.94 ± 1.81 ^a^
Taste	6.01 ± 1.96 ^b^	6.30 ± 1.68 ^b^	6.35 ± 1.43 ^b^	6.24 ± 1.65 ^b^	5.36 ± 1.92 ^a^
Overall acceptability	6.38 ± 1.50 ^b^	6.77 ± 1.19 ^c^	6.38 ± 1.21 ^bc^	6.39 ± 1.41 ^bc^	5.60 ± 1.37 ^a^

^1^ Chickpea flour; ^2^ Chestnut flour. Values with different letters in the same row represent statistically significant differences (*p* < 0.05).

## Data Availability

The data presented in this study are available on request from the corresponding author.
